# Supplementary material for: Skyrmion ratchet propagation: utilizing the skyrmion Hall effect in AC racetrack storage devices
Source: Sci Rep. 2021 Feb 4;11:3020. doi: 10.1038/s41598-021-81992-0 (PMC7862652; doi:10.1038/s41598-021-81992-0)
Supplement: Supplementary file 1 — Supplementary Figures [file 41598_2021_81992_MOESM1_ESM.pdf]

Supplemental Material for "Skyrmion ratchet propagation: Utilizing the skyrmion Hall effect in AC racetrack storage devices"

Börge Göbel[1,*] and Ingrid Mertig[1]

[1]Institut für Physik, Martin-Luther-Universität Halle-Wittenberg, D-06099 Halle (Saale), Germany

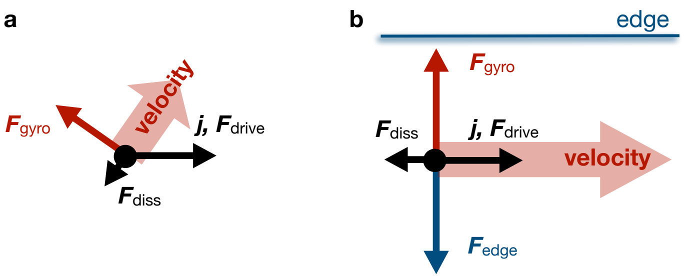

FIG. S1: **Forces acting on skyrmions.** **a** considers a free skyrmion and **b** show a skyrmion that creeps along the edge. Here, the edge force compensates the gyroscopic force and the net motion is parallel to the edge at an increased velocity compared to panel a.

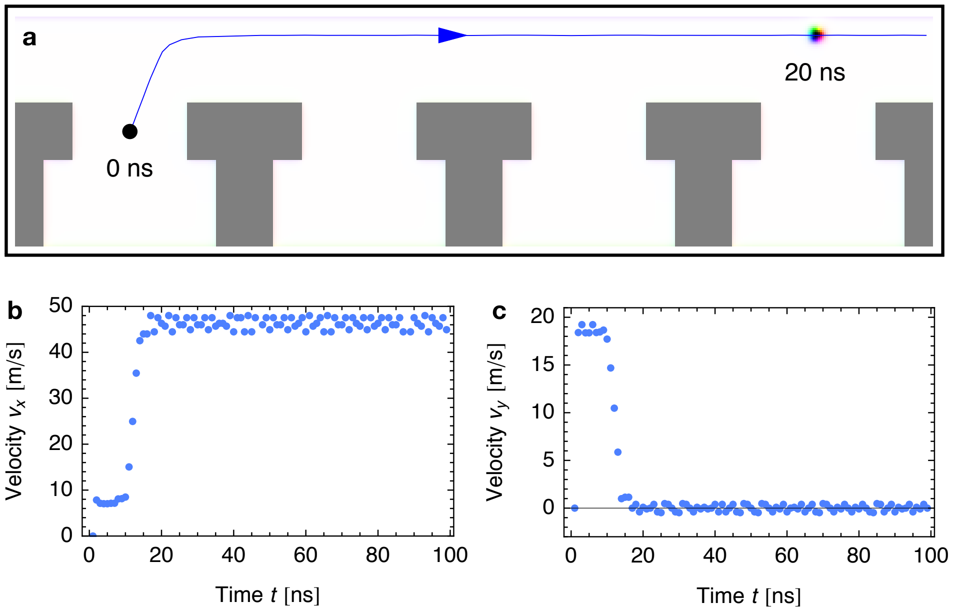

FIG. S2: **Skyrmion propulsion by a direct current.** **a** Trajectory in the geometry used in Fig. 6c but with a constant current of equivalent amplitude $j_{\mathrm{DC}}\Theta_{\mathrm{SH}} = 10/\sqrt{2}\,\frac{\mathrm{MA}}{\mathrm{cm}^2}$. **b,c** Velocity along the x and y directions, respectively.

* Corresponding author. boerge.goebel@physik.uni-halle.de
