# Supplementary figures and images for: Skyrmion ratchet propagation: utilizing the skyrmion Hall effect in AC racetrack storage devices
Source: Sci Rep. 2021 Feb 4;11:3020. doi: 10.1038/s41598-021-81992-0 (PMC7862652; doi:10.1038/s41598-021-81992-0)

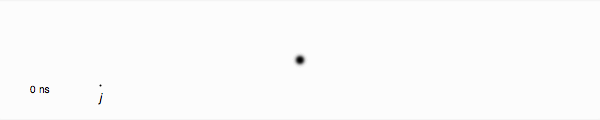

Supplement: Supplementary file 2 — Supplementary Movie 1 [file 41598_2021_81992_MOESM2_ESM.gif]

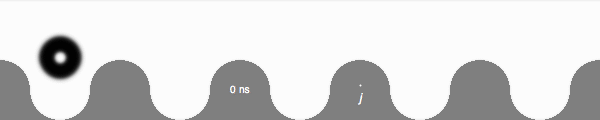

Supplement: Supplementary file 3 — Supplementary Movie 2 [file 41598_2021_81992_MOESM3_ESM.gif]

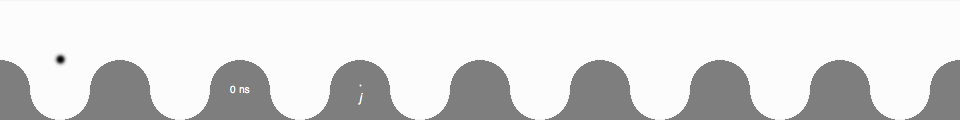

Supplement: Supplementary file 4 — Supplementary Movie 3 [file 41598_2021_81992_MOESM4_ESM.gif]

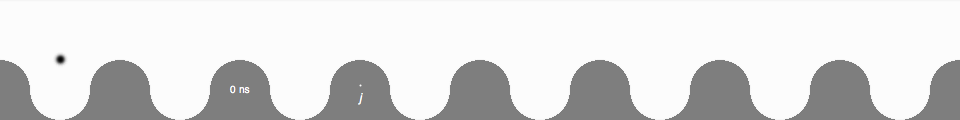

Supplement: Supplementary file 5 — Supplementary Movie 4 [file 41598_2021_81992_MOESM5_ESM.gif]

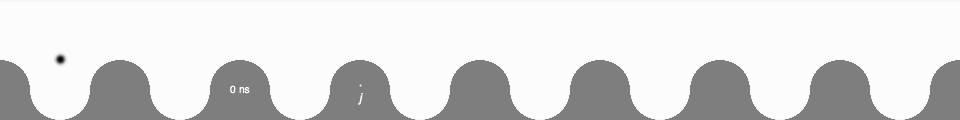

Supplement: Supplementary file 6 — Supplementary Movie 5 [file 41598_2021_81992_MOESM6_ESM.gif]

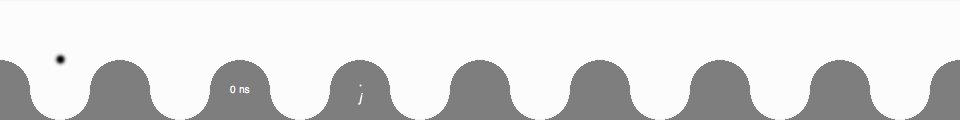

Supplement: Supplementary file 7 — Supplementary Movie 6 [file 41598_2021_81992_MOESM7_ESM.gif]

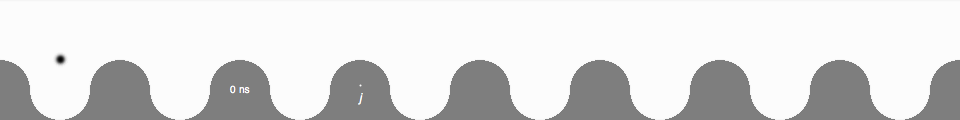

Supplement: Supplementary file 8 — Supplementary Movie 7 [file 41598_2021_81992_MOESM8_ESM.gif]

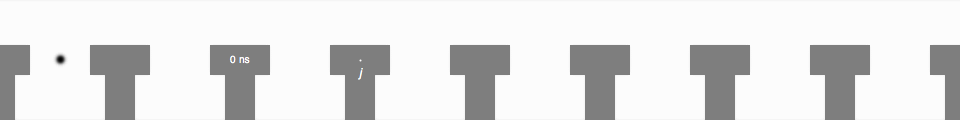

Supplement: Supplementary file 9 — Supplementary Movie 8 [file 41598_2021_81992_MOESM9_ESM.gif]

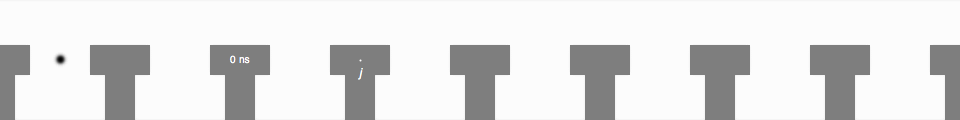

Supplement: Supplementary file 10 — Supplementary Movie 9 [file 41598_2021_81992_MOESM10_ESM.gif]

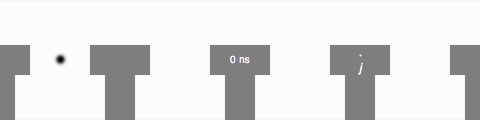

Supplement: Supplementary file 11 — Supplementary Movie 10 [file 41598_2021_81992_MOESM11_ESM.gif]
